# Time of Day Does Not Modulate Improvements in Motor Performance following a Repetitive Ballistic Motor Training Task

**DOI:** 10.1155/2013/396865

**Published:** 2013-03-14

**Authors:** Martin V. Sale, Michael C. Ridding, Michael A. Nordstrom

**Affiliations:** ^1^Queensland Brain Institute, The University of Queensland, St Lucia, Brisbane, QLD 4072, Australia; ^2^The Robinson Institute, School of Paediatrics and Reproductive Health, The University of Adelaide, Adelaide, SA 5005, Australia; ^3^Discipline of Physiology, School of Medical Sciences, The University of Adelaide, Adelaide, SA 5005, Australia

## Abstract

Repetitive performance of a task can result in learning. The neural mechanisms underpinning such use-dependent plasticity are influenced by several neuromodulators. Variations in neuromodulator levels may contribute to the variability in performance outcomes following training. Circulating levels of the neuromodulator cortisol change throughout the day. High cortisol levels inhibit neuroplasticity induced with a transcranial magnetic stimulation (TMS) paradigm that has similarities to use-dependent plasticity. The present study investigated whether performance changes following a motor training task are modulated by time of day and/or changes in endogenous cortisol levels. Motor training involving 30 minutes of repeated maximum left thumb abduction was undertaken by twenty-two participants twice, once in the morning (8 AM) and once in the evening (8 PM) on separate occasions. Saliva was assayed for cortisol concentration. Motor performance, quantified by measuring maximum left thumb abduction acceleration, significantly increased by 28% following training. Neuroplastic changes in corticomotor excitability of abductor pollicis brevis, quantified with TMS, increased significantly by 23% following training. Training-related motor performance improvements and neuroplasticity were unaffected by time of day and salivary cortisol concentration. Although similar neural elements and processes contribute to motor learning, training-induced neuroplasticity, and TMS-induced neuroplasticity, our findings suggest that the influence of time of day and cortisol differs for these three interventions.

## 1. Introduction

Learning a new motor task is associated with an improvement in performance. The neural adaptations to training that contribute to performance improvements depend on the type and duration of training. For example, learning to juggle daily for six weeks leads to structural changes in white matter density that persist for four weeks following cessation of training [1]. Shorter periods of training (30 mins) also lead to neural changes [2], but these changes are less enduring. Irrespective of the duration and type of training, motor learning can be divided into fast and slow learning phases, but the time involved in acquiring the performance gains in the different phases of learning is very much task specific [3]. Fast learning occurs after a single session of training, whilst slow learning occurs after several sessions, and involves “off-line” consolidation [4]. The relatively short time course involved in inducing training-related changes in cortical activity with fast learning makes these types of protocols particularly suitable for studying the neural adaptations to training, and is the type of learning involved in the present study. The neural changes associated with training also vary considerably between individuals. Some of the factors that influence an individual's neural response to training include genetic polymorphisms (e.g., brain-derived neurotrophic factor) [5, 6], attention [7], and age [8]. A key goal of neurorehabilitation research is to understand the mechanisms involved in mediating the effectiveness of training, thereby allowing for better targeted interventions. In this context, although repetitive training of tasks or movements is most obviously associated with rehabilitation for motor disorders, such as those induced by stroke, it also forms the basis of several rehabilitation interventions in cognitive neuropsychology, such as anxiety [9] and depression [10].

The development of various noninvasive brain stimulation techniques, such as transcranial magnetic stimulation [11], has provided the opportunity to more effectively probe neural adaptation to training in humans. Furthermore, experimental paradigms now exist that can induce changes in human cortex that modify neural pathways in a similar way to motor training. These paradigms involve either direct cortical stimulation [12–14], a combination of both peripheral and cortical stimulation [15–17], or repetitive peripheral stimulation [18, 19]. When targeted to the motor cortex, the same (or at least spatially very similar) circuits are modulated with these experimental paradigms as with repetitive motor training [2, 20–22]. Furthermore, the factors that are important in modulating training-related changes in performance (e.g., genetics, attention, and age) also influence how the brain responds to the various noninvasive brain stimulation techniques (e.g., [8, 23, 24]).

Time of day is one factor that has recently been shown to influence the magnitude of experimentally-induced neuroplasticity in human motor cortex. Using one such technique (paired associative stimulation; PAS) to induce plasticity within the human motor cortex [15], neuroplasticity induction was shown to be more effective and reproducible when experiments were performed in the afternoon [25] or evening [26] compared with the morning. The time of day modulation of neuroplasticity induction is due, at least in part, to changes in circulating cortisol levels [26]. This finding suggests that neurorehabilitation of motor function should be more effective if carried out in the afternoon, but the effect of time of day on training-induced neuroplasticity has not been investigated. We hypothesised that if the neural circuits that drive neuroplastic change following PAS are similar to those altered during motor training, then improvements in motor performance and training-induced neuroplasticity will be greater in the evening compared with the morning. We also predicted that these differences will be associated with changes in circulating cortisol levels.

## 2. Materials and Methods

### 2.1. Participants

Twenty-two right-handed participants (aged 19–37 years; 10 females) completed the study, which was approved by the University of Adelaide Human Research Ethics Committee. All participants had no known history of neurological conditions and gave written informed consent to participate in the study. Each participant attended two separate experimental sessions, once in the morning (8 AM) and once in the evening (8 PM). The experimental sessions were separated by at least one week, and the order was randomised. 

### 2.2. Recording

Surface electromyographic (EMG) recordings were made from left abductor pollicis brevis (APB) muscle. EMG signals were amplified (×1000), filtered (20–500 Hz), and digitized (2000 Hz) using a CED 1401 interface (Cambridge Electronic Design) and stored on computer for off-line analysis.

Acceleration recordings were made from the left thumb using a dual-axis accelerometer (ADXL311 Analog Devices Inc., MA, USA). The accelerometer was affixed to a splint that was taped to the second phalanx of the thumb, with its axes aligned to record abduction and flexion about the joint. Acceleration signals were amplified (×3), filtered (low pass 50 Hz), digitized (2000 Hz) using a CED 1401 interface, and stored on a computer for off-line analysis. Participants were provided with visual feedback about abduction and flexion acceleration for each trial and were encouraged to minimise flexion movements throughout the experiment. Flexion data were not further analysed.

### 2.3. Motor Training (MT) Task

The MT task involved rapid abduction of the thumb and has been previously described [2, 17]. The left hand was placed in a device that constrained the left forearm in mid-range supination and maintained the wrist in ~45 degrees extension. The left shoulder was kept in a neutral position (slight external rotation), and the elbow was flexed at ~90 degrees. The device allowed movement of the thumb in all planes. The MT task consisted of repeated abduction of the left thumb at maximal acceleration, paced by an auditory tone (1000 Hz tone, 100 ms duration) once every 2 seconds for 30 minutes. Participants were provided with performance feedback that consisted of visual display of maximum thumb acceleration on a computer screen for each trial, as well as verbal encouragement throughout the MT task. Such a training task has been shown to demonstrate features of motor learning such as long-term improvement in performance [20, 27] and the ability to interact with factors that influence learning [2, 20, 21]. It has, therefore, been used by several groups [2, 17, 20, 21, 27] as a model of (fast) motor learning. 

### 2.4. Quantification of Training-Induced Changes

#### 2.4.1. Maximum Thumb Abduction Acceleration

To quantify the effect of the MT task on motor performance, ten trials of maximal left thumb abduction acceleration were obtained prior to and following the MT task. In order for participants to familiarise themselves with the required task, they were allowed a few (<5) practice trials of the thumb abduction task at the start of the first experimental session only. 

#### 2.4.2. Transcranial Magnetic Stimulation (TMS)

Cortical excitability changes associated with the MT task were quantified using TMS. All participants completed a TMS safety screen [28] and were excluded if there was a family history of epilepsy, were taking any neuroactive drugs, or had undergone neurosurgery. Monophasic TMS was applied through a figure-of-eight coil (outer diameter of each wing 90 mm) which was connected to a Magstim 200 magnetic stimulator (Magstim, Whitland, Dyfed, UK). The coil was held tangentially to the skull with the handle pointing backwards and laterally at an angle of 45° to the sagittal plane at the optimal scalp site to evoke a motor evoked potential (MEP) in the relaxed APB muscle of the left hand. With this coil placement, current flow was induced in a posterior-to-anterior direction in the brain. The optimal scalp position was marked with a pen, and the coil was held by hand, with the position continually checked during TMS. Prior to MT, the TMS intensity required to produce MEPs with a peak-to-peak amplitude of between 0.5 and 1.0 mV was determined (SI_pre_). Ten MEPs using SI_pre_ were obtained prior to MT (0.2 Hz), and then again following MT. The cortical excitability data were obtained immediately prior to the maximum thumb abduction acceleration data for both before and after MT.

### 2.5. Experimental Protocol

Motor performance was assessed by measuring maximum left thumb abduction acceleration prior to (pre-MT) and five minutes following MT (post-MT). Thumb acceleration data were obtained immediately after MEP trials. Participants were given verbal and visual encouragement to perform their best possible thumb accelerations. There was no restriction on the frequency of thumb acceleration trials during the pre-MT and post-MT assessment periods.

Maximum thumb acceleration data were collected during the MT task and later analysed for rate of improvement in thumb acceleration and standard deviation of thumb acceleration during training (see Section 2.7).

Cortical excitability was assessed by measuring mean peak-to-peak amplitude of the APB MEP at rest. It was calculated by averaging the individual peak-to-peak amplitudes of MEPs elicited by 10 TMS (0.2 s^−1^, intensity SI_pre_) delivered immediately prior to (pre-MT) and five minutes following MT (post-MT).

### 2.6. Salivary Cortisol Assay

Saliva samples were collected from each participant prior to commencement of MT and at the end of each experiment. Saliva was frozen at −20°C until assayed. On the day of assay, the saliva samples were thawed and centrifuged. Twenty-five *μ*L of saliva was assayed in duplicate for cortisol by ELISA (HS-Cortisol; Salimetrics, LLC, State College, PA, U.S.A.).

### 2.7. Statistical Analysis

Separate two-way repeated measures analyses of variance (ANOVAs) were performed on thumb acceleration and APB MEP amplitude with within-subject factors intervention (two levels: pre-MT and post-MT) and time of day (two levels: AM and PM). 

To investigate changes in thumb acceleration during the MT task, data were divided into six separate five-minute epochs (0–5 mins, 5–10 mins, 10–15 mins, 15–20 mins, 20–25 mins, and 25–30 mins). Mean APB acceleration and the standard deviation (SD) of APB acceleration were calculated for the separate epochs. Separate two-way repeated measures ANOVAs were performed on the mean thumb acceleration and SD data with within-subject factors EPOCH (six levels: 0–5 mins, 5–10 mins, 10–15 mins, 15–20 mins, 20–25 mins, and 25–30 mins), and time of day. 

A two-way repeated measures ANOVA was performed on salivary cortisol concentration with within-subject factors intervention and time of day.

Linear regression analysis was used to assess a relationship between salivary cortisol concentration and changes in motor performance with training (max. acceleration post-MT/max. acceleration pre-MT) and change in cortical excitability (APB MEP facilitation: post-MT MEP amplitude/pre-MT MEP amplitude). The salivary cortisol concentration data were log transformed to improve homoscedasticity. The strength of the relationship was quantified by the coefficient of determination (*r* ^2^).

For all analyses, *P* < 0.05 was chosen as the significance level, and unless stated otherwise, all group data are reported as mean ± SEM. Bonferroni *post-hoc* tests for multiple comparisons were performed as appropriate.

## 3. Results

### 3.1. Motor Performance and Motor Training

**Figure 1:**
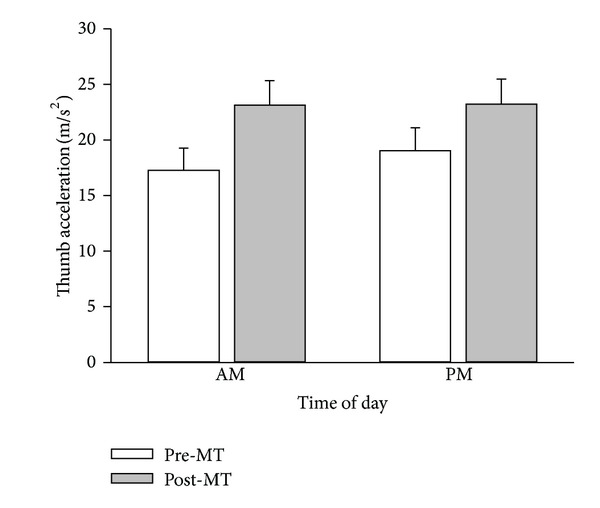
Improvement in maximum thumb acceleration after motor training is not influenced by time of day. Average (*n* = 10) maximum left APB abduction acceleration readings (mean ± SEM) recorded from 22 participants who participated in two experimental sessions: morning (AM) or evening (PM) on separate occasions. Average maximum thumb acceleration values are shown before (pre-MT) and after (post-MT) motor training. Maximum acceleration increased significantly following motor training, and this effect was independent of time of day.

A two-way ANOVA revealed that MT significantly increased maximum thumb acceleration by 28% (*F*_1,21_ = 22.61, *P* < 0.001; see Figure 1), but MT was not influenced by time of day (*F*_1,21_ = 0.284, *P* = 0.599). In addition, there was no significant interaction between intervention and time of day (*F*_1,21_ = 1.543) indicating that the improvement in thumb acceleration following training was independent of time of day. With 22 participants, an effect size of 0.241 (partial *η* ^2^ = 0.055) and with an *α* error of 0.05, the power to detect an effect was 0.74.

**Figure 2:**
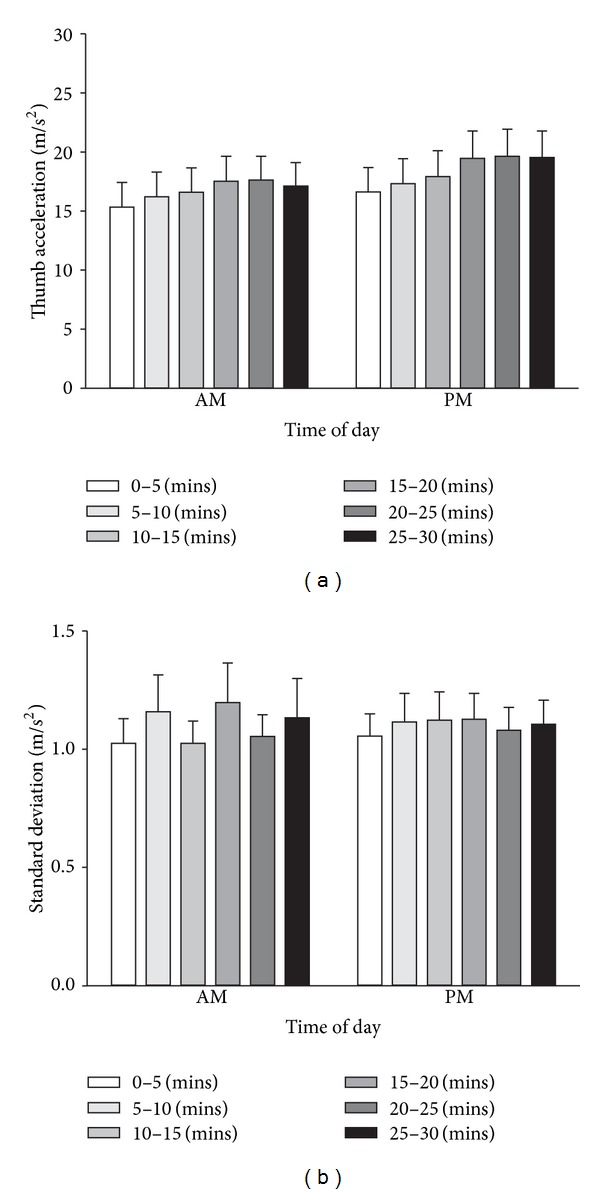
Time of day did not influence neither the maximum left thumb acceleration nor the standard deviation of maximal thumb acceleration during a motor training task. Average APB peak abduction acceleration (a) and standard deviation (b) values (mean ± SEM) were recorded from 22 participants during a motor training task on two separate occasions: morning (AM) and evening (PM). Data are divided into six 5-minute epochs for the motor training task (0–5 mins, 5–10 mins, 10–15 mins, 15–20 mins, 20–25 mins, and 25–30 mins). Acceleration increased significantly during MT, for both morning and evening sessions. The trial-to-trial variability of acceleration (SD) did not change during MT, and this effect was similar in morning and evening sessions.

Mean maximum acceleration values during the MT task are shown in Figure 2(a). Two-way repeated measures ANOVA revealed a significant effect of EPOCH (*F*_5,105_ = 13.01, *P* < 0.001). *Post-hoc* analysis revealed that mean maximum acceleration during the 0–5 minutes epoch was significantly less than at the 10–15 minutes (*P* = 0.003), 15–20 minutes (*P* < 0.001), 20–25 minutes (*P* < 0.001), and 25–30 minutes epochs (*P* < 0.001). Mean maximum acceleration during the 5–10 minutes epoch was significantly less than during the 15–20 minutes (*P* < 0.001), 20–25 minutes (*P* < 0.001), and 25–30 minutes (*P* < 0.001) epochs. Mean maximum acceleration during the 10–15 minutes epoch was significantly less than during the 20–25 minutes (*P* = 0.002) epoch. Motor performance during the MT task was not influenced by time of day (*F*_1,105_ = 0.885). The interaction term epoch × time of day was not significant (*F*_5,105_ = 1.322), indicating that the improvement in performance during the MT task was independent of time of day.

The variability of maximum thumb acceleration data during the MT task is shown in Figure 2(b). Two-way repeated measures ANOVA revealed no significant effect of epoch on the standard deviation of maximum thumb acceleration data during the MT task (*F*_5,105_ = 1.026, *P* > 0.05). The SD of acceleration during the MT task was not influenced by time of day (*F*_1,105_ = 0.003, *P* > 0.05). The interaction term epoch × time of day was also not significant (*F*_5,105_ = 0. 432, *P* > 0.05).

### 3.2. MEP Changes and Motor Training

TMS intensity that was used to evoke test MEPs was 5% higher in the evening (73.6 ± 2.8% MSO) compared with the morning (70.4 ± 2.4% MSO) (paired *t*-test, *P* = 0.037). Despite a time of day difference in test TMS intensity, pre-MT APB MEP amplitudes were not significantly different between groups (AM = 0.65 ± 0.07 mV; PM = 0.67 ± 0.07 mV; paired *t*-test).

**Figure 3:**
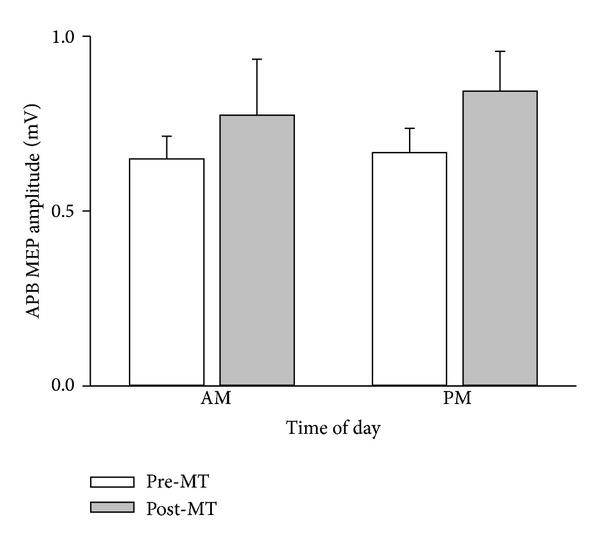
Increases in APB MEP amplitude after motor training are not influenced by time of day. Average (*n* = 10) APB MEP amplitude (mean ± SEM) recorded from 22 participants who participated in two experimental sessions: morning (AM) and evening (PM) on separate occasions. Average APB MEP amplitudes are shown before (pre-MT) and after (post-MT) motor training. APB MEP amplitude increased significantly following motor training, and this effect was independent of time of day.

Two-way ANOVA revealed a significant effect of motor training on MEP amplitude (intervention effect: *F*_1,21_ = 4.463, *P* = 0.047). APB MEP amplitude increased significantly by 23% following MT (Figure 3). There was no effect of time of day on MEP amplitude (*F*_1,21_ = 0.165, *P* = 0.688). In addition, there was no significant interaction between intervention and time of day (*F*_1,21_ = 0.086) indicating that the increase in MEP amplitude following MT was independent of time of day. With 22 participants, an effect size of 0.222 (partial *η* ^2^ = 0.047) and with an *α* error of 0.05, the power to detect an effect was 0.66.

#### 3.2.1. MEP Changes, Motor Performance, and Salivary Cortisol Concentration

**Figure 4:**
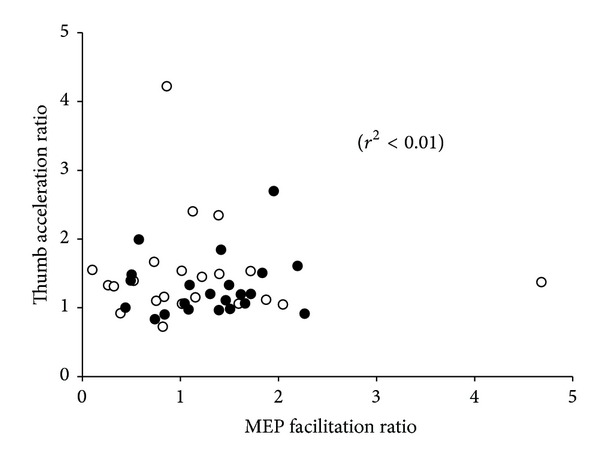
Relationship between motor performance improvement and cortical excitability changes following MT. Linear regression analysis revealed a nonsignificant relationship (*r* ^2^ < 0.01) between acceleration ratio (max. acceleration post-MT/max. acceleration pre-MT) and MEP facilitation ratio (MEP amplitude post-MT/MEP amplitude pre-MT). Data include morning (unfilled circles) and evening (filled circles) experiments.

Linear regression analysis between the changes in motor performance (acceleration) with training and the extent of APB MEP facilitation associated with training showed no significant relationship (*r* ^2^ < 0.01). These results are shown in Figure 4. 

**Figure 5:**
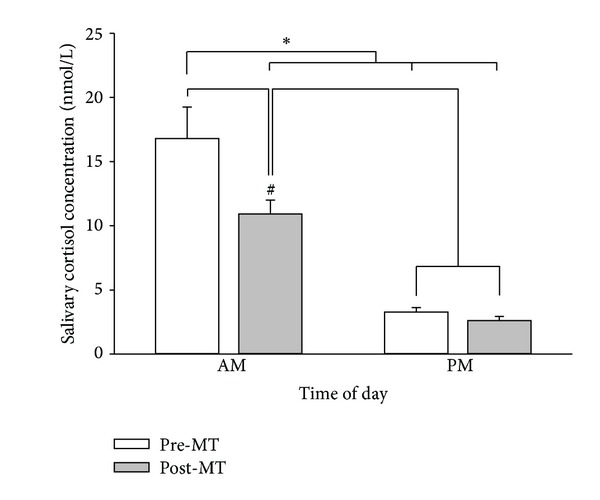
Salivary cortisol concentration is higher in the morning than the evening. Pre-MT salivary cortisol concentration in the morning was significantly greater than all other samples (*P < 0.001). Post-MT salivary cortisol concentration in the morning was significantly greater than both evening samples, but significantly less than pre-MT morning concentration (^#^ *P* = 0.008).

As expected, salivary cortisol concentration was significantly greater in the morning compared with evening experiments (*F*_1,21_ = 52.633, *P* < 0.001). Additionally, salivary cortisol concentration was significantly less following MT (*F*_1,21_ = 8.737, *P* = 0.008). The interaction term time of day × intervention was also significant (*F*_1,21_ = 7.477, *P* = 0.012. *Post-hoc* analysis revealed a significant reduction in salivary cortisol concentration following MT in the morning but not the evening experiments (*P* = 0.001). Salivary cortisol concentration data are shown in Figure 5. 

**Figure 6:**
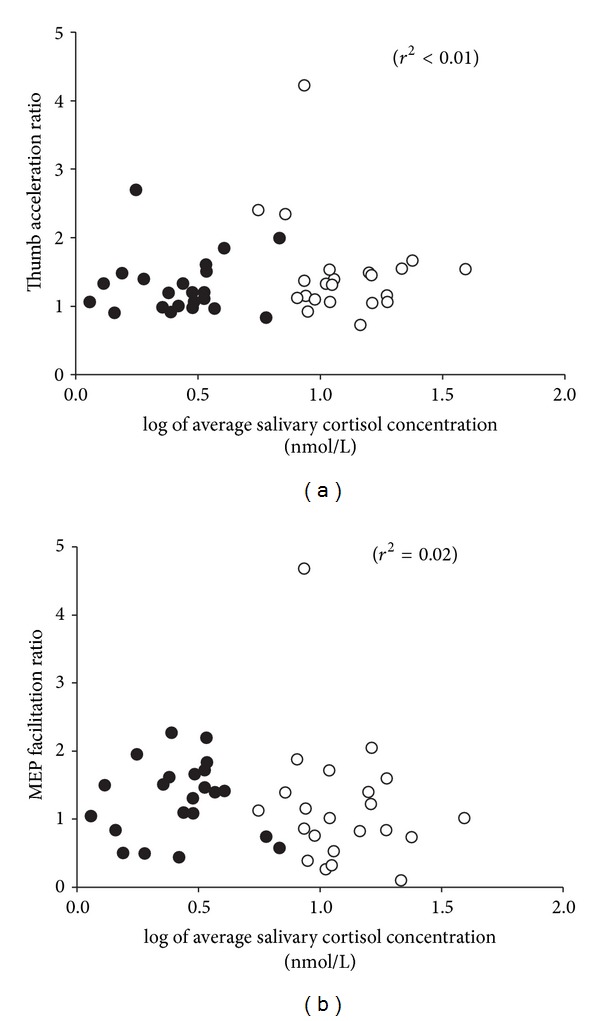
Relationship between motor performance (a), cortical excitability (b), and salivary cortisol concentration. Linear regression analysis revealed a nonsignificant (*r* ^2^ < 0.01) relationship between acceleration ratio (max. acceleration post-MT/max. acceleration pre-MT) and the log of average salivary cortisol concentration (a). Linear regression analysis revealed a nonsignificant (*r* ^2^ = 0.02) relationship between MEP facilitation ratio (MEP amplitude post-MT/MEP amplitude pre-MT) and the log of average salivary cortisol concentration (b). Data include morning (unfilled circles) and evening (filled circles) experiments.

Since salivary cortisol concentration significantly reduced over the time it took to perform the MT task in the morning, the pre-MT and post-MT salivary cortisol concentrations were averaged to provide a value that reflects the mean circulating cortisol level during MT. This was used in the linear regression analysis of the association between salivary cortisol concentration and both motor performance and the extent of APB MEP facilitation. There was no significant relationship neither between motor performance and the (log of) average salivary cortisol concentration (*r* ^2^ = 0.005; Figure 6(a)) nor between the extent of APB MEP facilitation and the (log of) average salivary cortisol concentration (*r* ^2^ = 0.016; Figure 6(b)).

## 4. Discussion

The principal finding from the present study was that the ballistic motor training task induced changes in motor performance and corticospinal excitability, but these changes were not influenced by time of day or circulating cortisol levels. Repeated maximal left thumb abduction for 30 minutes increased motor performance (indicated by an increase in maximal acceleration of thumb abduction) and induced an increase in corticomotor excitability of a thumb muscle used in the task (measured as an increase in APB MEP amplitude). However, the magnitude of the changes in both motor performance and cortical excitability were similar in the morning and evening sessions.

The present study was motivated by earlier research which showed that the effectiveness of plasticity induced in human motor cortex using paired associative stimulation is influenced by the time of day, with neuroplasticity induced more effectively in the evening compared with the morning [25, 26]. In addition, the effects induced with PAS were abolished when salivary cortisol levels were pharmacologically elevated, suggesting that cortisol levels mediate the effectiveness of PAS [26]. The PAS paradigm, initially described by Stefan et al. [15], repeatedly pairs a peripheral electrical stimulus with a later cortical (TMS) stimulus delivered to the contralateral motor region. The change in cortical excitability induced with PAS is believed to represent associative plasticity induced in motor cortex [15, 29]. This form of plasticity shares many similarities with use-dependent plasticity that occurs following training [2, 20–22]. For example, both forms of plasticity are thought to involve long-term potentiation-like changes in synaptic efficacy [15, 30]. Furthermore, PAS and motor learning interact in a homeostatic metaplasticity manner [2, 20–22]. Therefore, the present study sought to investigate whether use-dependent plasticity, induced following a period of motor training, is similarly influenced by the time of day of training. 

Performance of several motor tasks have been shown to be dependent on time of day [31]. For example, force discrimination [32], muscle strength [33], and performance of a basic motor flicking [34] and serial response task [35] are all influenced by time of day. However, none of these studies examined whether the learning of these tasks was influenced by time of day. Our results suggest that training-related improvements in motor performance (Figure 1) and cortical excitability (Figure 3) following motor training are neither influenced by the time of day nor by diurnal changes in circulating cortisol levels (Figure 6). 

Although no time of day differences in motor performance were reported following completion of the MT task, perhaps the rate of improvement in performance during the MT task was different in the morning and evening experiments. That is, although the magnitude of the improvement in motor performance was similar in morning and evening experiments, the improvement in performance in the evening experiments may have occurred more rapidly. McDonnell and Ridding [36] demonstrated that there were differences in the rate, rather than the overall magnitude, of improvement in a grooved pegboard task following afferent stimulation [36]. Analysis of the thumb acceleration data during the MT task revealed that motor performance improved significantly during the MT task, but the time-course of improvement in performance was similar in the morning and evening experiments (Figure 2(a)). 

Selective attention is important in modulating the effectiveness of neuroplasticity induced with TMS [23, 37]. If subjects were not consistently attending to the training task, we would expect to see greater trial-to-trial variability in thumb acceleration. We do not think that time of day differences in attention to the task influenced our results as there were no significant differences in the trial-to-trial variability of task performance for training conducted in the morning versus evening sessions (Figure 2(b)). 

There is good evidence that motor training increases cortical excitability in motor regions of the brain. For example, long-term learning of a sequential finger-thumb opposition task induces focal increases in blood flow to the primary motor cortex when participants perform the trained movements, as assessed with functional magnetic resonance imaging [38]. Moreover, a repetitive sequential finger training task, which improves motor performance (evidenced by reduced errors), results in an expansion of motor cortical maps of the practicing muscles [39]. Using a repetitive ballistic pinch training task, Muellbacher et al. [27] also demonstrated an improvement in motor performance following the training task, and showed that the improvement in performance was associated with changes in cortical excitability assessed with TMS. Several other studies have demonstrated such a relationship between cortical excitability and performance [37, 40, 41]. Since training-related changes in cortical excitability appear related to changes in motor performance, several studies have investigated whether an experimentally-induced increase in cortical excitability will influence motor performance. The results to date have been equivocal. Some studies have demonstrated a change in motor performance of the contralateral [42] and ipsilateral hands [43, 44] following rTMS. However, there have also been reports that an rTMS-induced increase in cortical excitability does not produce a change in motor performance [45, 46]. When the time course of changes in these two variables (cortical excitability and motor performance) was assessed following motor training. Muellbacher et al. [27] reported that whilst performance improvements remained 30 days after training, motor cortical excitability had returned to baseline. One possible explanation may be that the training-related neuroplastic change induced in the motor cortex, as evidenced by an increase in cortical excitability, may represent an early stage of motor learning [47] and that consolidation of that memory occurs in a remote area of the cortex. Also, individuals with a polymorphism in brain-derived neurotrophic factor, a gene known to be important in mediating neuroplastic change show performance improvements following training, but do not show any training-related change in cortical excitability as assessed with TMS [6]. Our results, showing no significant correlation between training-related changes in thumb acceleration and MEP amplitude, further support the notion that the interaction between performance improvements following training and changes in cortical excitability assessed with TMS is complex. We, therefore, contend that although similar neural circuits are activated and subsequently modified by motor training and various rTMS paradigms (including PAS), they are (a) not identical and/or (b) are differentially sensitive to inputs from other cortical/subcortical structures. The current experimental design does not allow us to distinguish between these options, but this could be addressed in future studies. 

### 4.1. Is Time of Day an Important Variable to Consider When Implementing a Training Regime?

The results of the present study suggest that time of day, and circadian variation in circulating cortisol levels, do not influence the effectiveness of short-term motor training. An important caveat of the present findings is that we do report a null effect. A power analysis undertaken on the acceleration and MEP data revealed that both data sets were somewhat under-powered (power = 0.74 and 0.66, resp.). With increased power, achieved by testing more participants, we could state with more confidence whether a time of day effect actually exists. However, we consider a time of day influence on performance and cortical excitability unlikely as the *P* value obtained for the critical time of day analyses for both performance (*P* = 0.599) and cortical excitability (*P* = 0.688) suggest that there is a high likelihood that time of day does not influence motor learning. In any case, our results suggest that the influence of time of day on motor learning is subtle, at best. This finding has implications in the cognitive domain, particularly when treatment or training involves a repetitive, use-dependent form of learning. Specifically, the results suggest that performance improvements should occur equally effective and irrespective of the time of day that training is undertaken. However, it is becoming increasingly common to augment repetitive (motor) training with repetitive peripheral and/or cortical stimulation paradigms [48–51]. Prior activation of targeted neural circuits with such paradigms may offer a way of priming cortical circuits, making them more receptive to subsequent training. We suggest that in these instances factors that influence the effectiveness of TMS-induced plasticity may, albeit indirectly, influence the effectiveness of subsequent training. In this situation, the previous findings of Sale et al. [25, 26] that time of day, and circulating cortisol levels, influence the effectiveness of TMS-induced plasticity in the motor cortex may be important. 

In conclusion, a repetitive ballistic MT task improves motor performance and increases motor cortical excitability that outlasts the training period. However, the magnitude of these changes is not influenced neither by the time of day nor by physiological variations in endogenous salivary cortisol concentration. These findings are of importance to clinicians and researchers using therapies reliant on use-dependent plasticity to improve function. They suggest that the magnitude of improvements seen with repetitive training tasks is similar for training conducted in the morning and evening.
